# Individual Variation in Perception and Evolutionary Aesthetics: Grant Allen's 1878 Investigation of Congenital Amusia

**DOI:** 10.1002/jhbs.70078

**Published:** 2026-11-12

**Authors:** Marjorie Perlman Lorch

**Affiliations:** ^1^ School of Creative Arts, Culture and Communication, Faculty of Humanities and Social Sciences, Birkbeck University of London London UK

**Keywords:** 19th century, aesthetics, experimental psychology, human evolution, music processing

## Abstract

The consideration of human evolution and variation were live topics in the second half of the 19th century. These issues converged in Grant Allen's (1848–1899) 1878 investigation of a case of “note‐deafness” (i.e., tone‐deafness; congenital amusia). This represents an early and overlooked example of experimental research into a selective perceptual deficit in an otherwise typically developing person. Allen employed objective tests to determine that the participant could hear but not accurately discriminate between pitches, while demonstrating preserved auditory perception of timing and intensity. He documented the participant's exposure, motivation, and training opportunities, and evidence of poor musical ability in family members. Parallel aspects of music and speech were investigated in perception and expression, and the neurological source of these difficulties was considered. These elements are hallmarks of high procedural rigor. Allen made connections between this music processing difficulty and his broader interests in the evolutionary physiology of aesthetics. Although Allen's study began to be cited in the 21st century, it has received little detailed consideration. This article examines the context and motivation for Allen's work and subsequent developments in the understanding of individual differences in sensory perception and processing. The interrelation between physiological variation and cultural practices that informed Allen's research questions regarding this selective perceptual impairment continue to figure in our view of congenital amusia and other individual differences. This article examines Allen's research questions as formulated in the late 19th century and those that remain unresolved in the understanding of human variation in perceptual processing.

## 1 Introduction

Consideration of musical ability has held a significant position in the natural sciences and philosophy in relation to the human capacities for emotional expression, spoken language, dancing, and other cultural artifacts (Cross 2001). In the second half of the 19th century, philosophers and scientists made efforts to develop an understanding of psychology within the frame of natural evolutionary processes (Draper 1863). Music has figured in theories about human evolution regarding the origin of language, the nature of mental faculties, and the neurological basis of motor expression and auditory perception (Kleinman 2015). There was increasing interest in the status of music following the major contributions in biological, psychological and social theories from the philosopher Herbert Spencer (1820–1903) (1855, 1857), the scientists Charles Robert Darwin (1809–1882) (1871, 1872) and Hermann von Helmholtz (1821–1894) (1863/1875) and the psychologist Edmund Gurney (1876).

A single case study published in 1878 by Charles Grant Blairfindie Allen (1848–1899), known as Grant Allen, appears to be the first systematic investigation of an individual with congenital amusia (Allen 1878b). Allen's research was conducted in the context of British debates about human evolution and individual variation. He was a strong supporter of both Spencer and Darwin and was well acquainted with the psychological issues and evidence being discussed in the mid‐19th century. Unlike earlier descriptions of people lacking in musical ability, Allen used rigorous systematic testing to investigate various aspects of his participant's perception of sound and music and was careful to objectively frame his interpretation of the evidence. In this, his investigation was more akin to the empirical style of Darwin, which relied on observations and measurements rather than the philosophizing of Spencer. Although there was limited understanding of the acoustic and physiological nature of audition at the time, Allen drew on the state‐of‐the‐art research by Helmholtz that had been recently translated from German into English (Helmholtz 1863/1875) for his interpretation of the case. He also drew an analogy between the relatively well‐developed understanding of color blindness, which was known to be predicated on hereditary and physiological factors (Wilson 1855), and this new limitation in music processing.

Allen was original in synthesizing a range of contemporaneous ideas on human evolution, culture and physiology as sources for the interpretation of musical processing. This case study was part of a larger research project that he initiated in the late 1870s into evolution and sensory aesthetics (Allen 1877, 1879a). Although Allen's case is now typically given priority as the first record of what is now called congenital amusia, some commentators have dismissed it for being anecdotal (e.g., Ayotte et al. 2002), and there has been little historical examination of this work. Allen presented a well‐developed research strategy and sophisticated approach to the empirical testing of the perception, discrimination and identification of pitch and pitch sequences in music and speech have been overlooked. It is argued here that this valuable contribution to early British experimental psychology should be recognized.

The first section of this article details the historical background of Allen's study regarding new ideas about human evolution and variation and their consequences for the appreciation of music that developed in the 1850s to 1870s. The next section provides context by examining the biographical details of Allen's education and early career, his intellectual influences and social network. Allen (1878b) single case study of congenital amusia is examined in regard to his methods, findings, and interpretation. Finally, the article considers the initial responses to Allen's case study and later developments to understand why this insightful and sophisticated work had a limited impact although it was published in a highly regarded journal and he had a strong network of influential supporters that included Darwin and Spencer. Allen's case attracted sporadic interest with respect to developmental and acquired music processing impairments in the following decades but detailed investigations into congenital amusia were relatively rare until the early 21st century (e.g., Peretz et al. 2002).

The significance of this investigation is in the identification of an early experimental psychology study that has been neglected. The article presents a detailed examination of the individuals and disciplinary orientations that were active in the 1870s and how Allen's work intersected with leading scientific questions and cultural issues of the day. Most histories of psychology identify the work of the German scientist Wilhelm Wundt (1832–1920) and those in his lab in Leipzig as the instigators of this type of research (e.g., Robinson 1995). The initiation of such research in Britain is typically not identified until almost a decade after its inception in Germany (Green 2009). Allen's case study is an overlooked piece of research which was an important originator of this methodology.

The consideration of Allen's work has been primarily focused on literary studies (e.g., Morton 2001; Rodgers and Greenslade 2005). This is understandable given that most of his well‐known publications are forms of fiction. His “popular” science writings do make up a substantial portion of his voluminous output (see Morton 2005b), but they have received less attention from historical scholars. In addition to Allen (1878b) experimental case study on music perception and processing, he wrote two substantial monographs on evolutionary aesthetics in the late 1870s (Allen 1877, 1879). Allen's work in this period focuses on topics which have great currency in research today. This article presents a detailed examination of Allen's contributions into individual variation and its evolutionary and cultural sources and their significance for the understanding of these issues today.

## 2 Background and Context

### 2.1 Music Aptitude and Cultural Activity

Music is a culturally shaped activity whose practice may be restricted to an elite group or more widely integrated into the public life of a community. It is often recognized as a talent that is unequally distributed in the population and valued as such. Currently, there are a relatively large minority of people who self‐identify as “tone deaf,” however, for some of these individuals this may be due to negative early experiences of musical performance or lack of musical training. When examined more closely, this group of unmusical people seems to be distinct from a much smaller group who exhibit more extreme difficulties (Cuddy et al. 2005). In such people, this often involves difficulties in recognizing familiar melodies, noticing erroneous notes, or changes to a tune. In terms of expression, there may be problems in marking the beat, dancing to a rhythm, singing on key, or learning to play a musical instrument (Wise et al. 2007). In contrast, such people typically have no difficulties recognizing other nonmusical sounds or voices or appreciating the changes in pitch employed in speech intonation. The exploration of musical aptitude has been considered since antiquity (Humphreys 1993). Although the primary of focus has been on people with high musical ability, anecdotal descriptions of individuals with difficulties performing basic musical tasks have been recorded throughout history (e.g., Willis 1684; see Lorch 2010).

In the early 19th century, new theories were developed to understand different mental capacities and how they related to areas of the brain. In his efforts to provide such an account, Franz Joseph Gall (1758–1828) viewed music as a key faculty of the brain and an innate talent present in humans and animals alike (Eling et al. 2017). As the century progressed, ideas about the faculty of music continued to develop. During the first half of the 19th century there was growing public performance of music and appreciation for it by a wider range of social groups beyond the elite (Jackson 2019). Acoustic science and its relation to the physiology of sensation were topics of scientific and social interest in Victorian England (Draucker 2018). Public lectures on acoustics were delivered at the Royal Institution in London in the 1860s and 1870s by Helmholtz and the Irish physicist John Tyndall (1820–1893). Interest in the human capabilities for music was also evident in the medical sphere at this time. For example, the London physicians began to report cases of children with preserved musical abilities who were unable to express themselves in speech (Lorch and Greenblatt 2015). At the same time, aspects of human variation were being investigated by the early British Anthropologists (e.g., Tylor 1865, 1871) as part of wider conceptual developments in the evolutionary principles evident in biological processes.

The early work on individual variation in accounts of evolution theorized about the role of natural and sexual selection, adaptation, and survival of the fittest. There was a significant shift when Darwin (1871, 1872) proposed that the evolutionary forces which were active in plants, insects and animals also applied to human behavior. This was initiated by considerations of the heritability of exceptional mental abilities and talents, including music (Galton 1869). The evolutionary relation between music and speech was also considered. For example, Spencer (1857) reflected on how the cadence of the voice in music and speech was a product of both emotion and intellect, while Darwin (1871) proposed that music was the source of human language. Grant Allen was immersed in these intellectual debates and his work on note‐deafness can be seen as a direct response to the various ideas discussed above. Allen was an early devotee to evolutionary theory, adopting this worldview wholeheartedly as a young student. The next section details Allen's academic training and the early professional experiences that framed his work on note‐deafness.

### 2.2 Grant Allen's Academic Background and Early Career

Allen was born in 1848 to Joseph Antisell Allen, a Protestant minister and briefly Professor at Queen's University in Kingston, Ontario and Catharine Ann Grant (Cotton and Arsdel 2016). Allen had the opportunities of an elite education and appears to have been a gifted student (Morton 2005a). The family moved several times after leaving Canada when he was a young teenager, and he continued his studies in several countries. He had a tutor from Yale University in New Haven, then studied at the Collège Impérial in Dieppe, France, and at King Edward's School in Birmingham, England. Allen won a postmastership (academic scholarship) to Merton College, University of Oxford in 1867 where he earned a BA degree in Classics (Morton 2005a). After graduating from Oxford, he worked for a short while as a private tutor and schoolmaster in several preparatory colleges in England. He then moved to Jamaica to take up the post of Professor of Mental and Moral Philosophy and subsequently became Principle of a newly founded Government College in Spanish Town. He lectured on the ideas of Alexander Bain (1818–1903) and Herbert Spencer who had written influential books on psychology in the 1850s (Bain 1855, 1859; Spencer 1855). The demands of the college at Spanish Town were not extensive, and this allowed Allen time to read and develop his scientific and philosophical interests. His activities included human brain dissection (Morton 2001), which can be taken as an indication of his early efforts in empirical observation. Allen's reading on evolution and human behavior while in Jamaica would provide theoretical underpinning to his subsequent research investigations. When the Jamaican college closed in 1876, Allen returned to England. He set about establishing himself in scientific circles as a comparative psychologist and proponent of evolutionary theories.

Allen's own ideas are an eclectic blend of natural selection, survival of the fittest, adaptation, the influence of the environment, and cultural transmission. Allen also considered that aesthetics was an evolutionary driver of behavior in the natural world. At this time, the human response to beauty was a question of interest for the leading intelligentsia in the art world and reflected in the English Pre‐Raphaelite movement of the mid‐19th century. For example, the widely influential art historian John Ruskin (1819–1900) began writing about the wider aspects of aesthetics in the 1840s. This involved a consideration of how to conceptualize the human mind in relation to the religious and moral views in Christian‐based thought (Hewison 1976). In a discussion of the classical Greek literature on beauty, Ruskin (1877/1891) considered how the term “amusia” referred to the denial, or desolation for want, of the muses. Although the term later became used to refer to impairments in music perception and production (as discussed below), Ruskin's commentary is a signal of the wider interest in aesthetics that was prominent in the period that Allen was working (see Wilhelm 2016).

Following Darwin's and others' contributions in the early 1870s, there was a shift in thinking that viewed human behavior as a product of anatomy and physiology and subject to the forces of evolution rather than being a spiritual endowment. This included considerations of music and beauty as significant human behaviors formed by evolutionary forces (Radick 2013). Allen's work in the 1870s was situated in this school of thought. When the psychology journal *Mind* was founded in 1876, it included aesthetics as one area of interest (Staley 2009). Allen's (1878b) case study of music perception published in this journal intersects with this theme.

From 1876, Allen had begun writing science articles for many of the leading English periodicals of the day, including *Mind* (Morton 2005b). *Mind* was the first journal in the Anglophone world that specifically focused on publishing new research in philosophy and psychology. It began publication only a few years after the leading science journal *Nature* first appeared. Bain was its founder and George Croom Robertson (1842–1892), Professor of Philosophy of Mind and Logic at University College, London was the editor. The journal's initial aim was to determine whether the new field of psychology in Britain could be based on experimental research as it was beginning to be in Germany. This contrasted with the predominant scientific method that employed naturalistic observation and introspection as represented by the work of Darwin and Spencer (Green 2009). Up until this point, scientists in Britian had done little in the way of experimental investigations on psychological issues. Wundt's lab in Germany, established in 1879, is typically viewed as the initiator of such methodological innovations (Robinson 1995). The following sections present evidence to argue that Allen's work has been overlooked as an early and significant contribution in British experimental psychology, to the understanding of music processing and the wider field of evolutionary aesthetics.

## 3 Allen's Research on Perception

### 3.1 Physiological Æsthetics

Before his case study appeared in *Mind*, Allen's published his first book, *Physiological Æsthetics* (Allen 1877). This presented a detailed account of how sensory preferences evolve in insects, birds, animals, and humans. Allen stated that his theme was motivated by Darwin (1871). He acknowledged the influence of Bain on his ideas, and he dedicated the book to Spencer. By this time, both Darwin and Spencer had become personal acquaintances and enthusiastic supporters of Allen's scientific endeavors (Rodgers and Greenslade 2005). Allen's book (1877) presented a physiologically based account of aesthetic sensibilities through consideration of each of the senses that are engaged in these responses. He analyzed aesthetic reactions throughout the natural world and accounted for them in terms of the neurophysiology of perceptual systems.

Allen (1877) argued that differences of sensation and perception depended upon differences in physical structure and drew on examples from animal and human variation. He pointed out the fact that people who are deaf or blind are unable to have any aesthetic experience of sound or color. He then argued:

Descending to cases of slighter variations, the colour‐blind cannot derive the same pleasure as others from judicious combinations of tints; and those utterly destitute of musical ear cannot be gratified or offended by harmony or discord. It is hardly necessary to point out that these differences depend upon abnormal nervous structure. Proceeding to matters of greater detail, all the senses yield us instances of varieties of Taste which are similarly referable to slight morphological peculiarities.

Allen 1877, 43

One relevant limitation to Allen's consideration of sensation and perception was that aspects of human hearing were not well understood at the time in contrast to human vision. Moreover, the selective visual perceptual impairment of color blindness had been a topic of active physiological and psychological inquiry for several decades. This condition was initially described by the English scientist John Dalton (1766–1844) in 1794 and investigated in detail by the Scottish chemist George Wilson (1818–1859) in 1855. Wilson's experiments had great impact due to the socio‐economic significance of recent high‐profile rail and maritime accidents that occurred due to the misapprehension of the color of signals. Wilson's objective was to identify people who should not be employed due to their difficulty in discerning the different signal colors (Wilson 1855). He determined that this relatively rare inability to see certain colors was hereditary and had a physical basis in the retinal fibers. A minority of people were selectively impaired in this ability but were otherwise typically abled. This was a new recognition of human variation in which there is impairment of selected perceptual capacities in otherwise healthy people.

Allen was aware of the evidence that the cause of color blindness was directly attributed to inherited limitations in the sensitivity to colors in the retina. He extended this observation in the visual domain to raise questions about its similarity to the difficulties in those who were limited in the ability to perceive consonance and dissonance in audition and to aesthetic appreciation more generally:

We found in Hearing that the peculiar fact upon which consonance and dissonance were based was that of interference, and the consequent production of beats; we have now to enquire what is the corresponding physical fact upon which is founded the feeling of harmony and discord in the perception of colour.

Allen 1877, 160

Allen (1877) further argued that experience and training had a minimal effect on the development of aesthetic sensibilities:

To employ the extreme illustration used above, the colour‐blind cannot possibly be taught to enjoy dainty contrasts of tint, nor the note‐deaf to be offended at musical discords. In every department the aim of Education should be so to train each individual that he may use to the best advantage the organism which heredity and circumstances have given him. More than this it cannot possibly effect.

Allen 1877, 50

These issues would also be pursued in his experimental case study of note‐deafness (Allen 1878b) discussed in detail below. It is not clear whether Allen was already considering this research project or had in fact initiated it at the time the book was written, as it has proved difficult to identify the participant and timing of the study.

In the chapter dedicated to hearing, Allen (1877) cited extensively from Helmholtz's *On the Sensation of Tone as a Physiological Basis for the Theory of Music* (Helmholtz 1863/1875). This work significantly contributed to the understanding of sound as a physical force and hearing as a physiological capability. Allen discussed the role of the multitude of fibers that arise from the organs of Corti to the higher nervous system. He described the different physiological mechanisms for processing noise and other sounds. He linked the perception of pitch and intensity to the nature of the fibers in the auditory nerve. Allen distinguished these objective stimuli from the subjective auditory phenomena of musical timbre and note. In his presentation of music, Allen (1877) defined the note in terms of the relation between the different tones that comprised an octave. These considerations figure in Allen (1878b) investigation of congenital amusia published a few months later.

### 3.2 Allen's Investigation of *The Color Sense*

During the period from the mid‐1870s onwards, Allen was also engaged with another research project. Although Allen's second monograph, *The Colour‐Sense: Its Origin and Development* was not published until 1879, he explained in the preface that he had been working on this project for several years. This focused study of the evolution and adaptation of color and color perception in fruit, flower, insects, birds, animals and humans had been initiated while he was in Jamaica. However, the project greatly expanded over time as Allen subsequently determined to collect evidence to respond to a lively, high‐profile public debate that was unfolding. The German ophthalmologist Hugo Magnus (1842–1907) had put forward the premise that human color vision had evolved in historical times (Schöntag and Schäfer‐Prieß 2007). This academic proposal gained more widespread consideration when William Ewart Gladstone (1809–1898), the British Prime Minister (1868–1874) and polymath, argued in favor of Magus's theory in the periodical *The Nineteenth Century* (Gladstone 1877). Gladstone supported this claim by pointing to the observation that the ancient Greek poet Homer showed little awareness of color in his writings. Several naturalists, including Allen's supporter Alfred Russell Wallace (1823–1913), had argued against this, and Allen determined to gather the factual evidence.

Allen's argument focused on the hereditary nature of variation and adaptation of sensation and perception in vision and aesthetics. In his prefatory remarks (Allen 1879), he makes it clear that botanists and zoologists were the source of the facts he used as evidence to support his arguments about natural and sexual selection. Allen had published a brief note in January 1878 in *Mind* putting forth his reasoning about the evolutionary basis of the color sense (Allen 1878a). To collect his evidence, Allen sent inquiries to people in many parts of the world to gather observations and he consulted thousands of references (Allen 1894). In investigating the claim, Allen used similar methods of direct observational evidence from his own travels and the collection of data from others through correspondence akin to those used by Darwin (Rodgers and Greenslade 2005). For example, he published a request for assistance in the *Journal of the Royal Anthropological Societ*y 1878). This detailed a set of questions to be asked about the color sense in people around the world. In this questionnaire, Allen's items differentiated the ability to perceive colors from having different names for them. He argued that this was a significant distinction that others had not taken care to untangle (Allen 1878c).

A further indication of his efforts to collect objective data is seen in an experiment briefly described in *Mind* (Miscellaneous 1879, 299). This was in response to assertions by the German anthropologist Adolf Bastian (1826–1905) and Gladstone that Burmese people were unable to distinguish between blue and green. Allen selected samples of blue and green colored paper which were of the same shade, intensity, and texture. He sent them to the British Resident in Burma Henry Lewis St. Barbe (born Henry Lewis St. Barbe Browne (c.1849−1886) and asked that he test peoples' ability to match them by color and name them. The people that were tested were described by St. Barbe as Burmese, Shans, Maingthas, Chinese, and Kachyens. He reported that none of the people he tested had any difficulty in matching the colored pieces. However, there was variation in the color names that were used depending on what language people spoke. Allen concluded that this was not a difficulty in perception but rather “insufficient vocabulary” (Miscellaneous 1879). Allen argued that color vision in insects, animals and people and the colors of flowers and fruits had evolved interactively. Furthermore, he suggested that color words developed in different cultures due to the range of pigments found in their environment rather than from a perceptual limitation. This interpretation of his results reflects Allen's wider interest in the interplay between genetic and cultural factors in sensory perception and aesthetics.

His book is subtitled “an essay in comparative psychology” and this point is highlighted in his preface (Allen 1879). Allen insisted that he was a comparative psychologist rather than a biologist. This indicates that at this time in his career Allen wanted to align himself with this new discipline. During the late 1870s when he was writing his monographs on aesthetics and evolution Allen published his first and it appears his only significant experimental research article (Allen 1878b). This is a single case study of an individual with impaired music processing. It was conducted at the request of the participant with testing that occurred over a period of months. The next section presents a detailed examination of Allen's data collection procedures, analysis, and interpretation.

## 4 Allen's 1878 Investigation of Note‐Deafness

In the introduction to his paper, Allen suggested that there was an “auditory abnormality” that was analogous to color blindness “…which I believe to be at least equally common, yet of which I have nowhere seen any definite account” (Allen 1878b, 157). Although this provides a clear rationale for his research, Allen's careful language leaves open the nature of the similarity between these two difficulties. As a wider motivation for his investigation, Allen stated: “All psychologists are now agreed upon the necessity for a greatly extended study of individual peculiarities” (Allen 1878b, 158). He also asserted that his work on this condition was original and that he had coined the term note‐deafness. In attempting to verify his claim of priority using present‐day techniques, a Google Scholar search was conducted. This is not a definitive record as many gaps in the database exist, nevertheless, only one study was identified through this search that predates Allen's on the empirical testing of music perception ability.

Three years earlier, the Glasgow physician John Gray M'Kendrick (1841–1926) published a paper in the *Proceedings of the Royal Society of Edinburgh* (M'Kendrick 1875). M'Kendrick examined the perception of music in 10 self‐selected individuals who professed to be unmusical. He tested his participants' ability to judge whether particular compound resonance made by forcing air through a long wooden box containing reeds was the same sound as a note played on a piano. He reported that nine of his participants could make this judgment correctly, while the tenth had difficulty with higher pitches, which appeared to be due to a hearing impairment. M'Kendrick argued that this demonstrated that people who reported having to be unmusical were capable of this analysis. He also investigated the more general ability to appreciate consonance and discord and intensity, which had been recently described by Helmholtz (1863/1875). M'Kendrick provided a detailed description of three sets of musical stimuli that increased in complexity. He reported that he himself and eight of his participants could hear the primary resonance but had difficulty with the secondary and tertiary tones. He mentioned the performance of two individuals with musical ability was better on stimuli with lower intensity. However, he did not provide systematic details of the performance of these two groups on each set of stimuli. This study provided detailed descriptions of the stimuli used in his two tests; however, the analysis of results and discussion of their implications was limited. In contrast to M'Kendrick's earlier report, which tested participants with varying musical ability on two tasks of resonance, Allen (1879b) presented a detailed case study of a participant with significant difficulty in pitch discrimination and appreciation of music. He collected data on a range of different variables and tested these systematically through a range of well‐described tasks.

The motivation for Allen's (1879b) case study is unclear. His particular interest in note‐deafness seems to have been purely academic. It was part of his larger interest in human sensory variation and the physiological sources of aesthetic sensitivities. There is an absence of biographical details pointing to Allen's personal involvement with music appreciation or performance. However, in this experimental case study, he acknowledged the assistance of two British scientists‐Francis Galton (1822–1911) and George John Romanes (1848–1894). Galton had written *Hereditary Genius* (1869), which put forth the argument that both physical and mental abilities are inherited by humans, an idea later developed by his cousin (Darwin 1871). Subsequently, Galton detailed the talents that ran in families, including music (Galton 1874). However, he himself was known to have disliked music and had unmusical relatives (Pearson 1930). Allen did not share the research perspective of his senior collaborator. While Galton was interested in the heritability of exceptional abilities as an indicator of high intelligence, Allen's study was about a selective lack of them in a highly intelligent person. Allen's peer Romanes was the only one involved in the study with a high appreciation of music and musical family members (Romanes 1896).

Allen's unnamed participant was a 30‐year‐old man of his acquaintance. He is described as “sufficiently educated to comprehend and answer in psychological terms all inquiries made of him, and with a competent knowledge of the physio‐logical mechanism of hearing.” (Allen 1878b, 157) In reporting this, Allen is establishing the participant's ability to understand and respond to the tests. The anonymity of the participant was maintained. No clues have been located in Allen's correspondence, archival materials, or biographical studies that shed light on his identity. All that is known is that he was the same age as Allen, had academic training and an interest in psychology, anatomy and physiology, and was likely based in London or nearby.

Allen appreciated that a lack of musical ability might be due to personal, social or environmental factors and took a case history to rule out the possibility that these were the source of his participant's difficulties. The participant reported a lack of interest in music since childhood. He had had unsuccessful experiences of singing with his family as a child and a failed attempt at formal music instruction when he was 16 years old. Allen offered these points as evidence of the individual's motivation and opportunity to develop musical skill. When the participant was older, he had attempted his own self‐experimentation to understand acoustic theory. He had tried unsuccessfully to learn the notes on the piano. He also employed instruments used to investigate how frequency relates to pitch, including a Savart's wheel and Robison's siren, to explore his own abilities psychometrically. From these experiences, the participant drew the conclusion that his own auditory abilities were significantly different from others. This motivated him to have Allen further examine his musical perception in a laboratory setting.

Allen (1878b) provides many background details about the participant which he regarded as relevant to the issue of pitch discrimination in music. He observed that the participant was a bad mimic of other people's speech patterns and regional varieties and spoke French which he had learned as a child with a pronounced English accent. However, Allen attributed these limitations to a motor production problem rather than a perceptual one. He also noted that the participant spoke in a monotone, which was not modulated to convey emotions. Moreover, Allen observed that if music was played in his presence, the participant appeared inattentive to it as a source of either positive or negative stimulation. The inclusion of these details suggests the various domains and variables that Allen considered to be relevant components of musical ability.

Allen sent a set of questions to the participant's family to ascertain their musical abilities and practices (Allen 1878b). This line of inquiry was likely to have been directly motivated by observations that color blindness was inherited, the recent studies by Galton, and Allen's more general interest in the evolutionary mechanisms for individual characteristics proposed by Darwin and Spencer. The participant's family reported that the father was able to distinguish between two adjacent notes on the piano but couldn't perceive any special relation between a tone and its octave. The brother was also described as unmusical but could tell if a singer was out of tune. In contrast, the mother and sisters were reported to be fond of music and sang and played instruments. Some more remote family members were also described as unmusical. These details indicate that the participant's male relatives may have experienced musical processing difficulties. However, it is not known whether this reflected a bias in social roles and opportunity or congenital impairment. Allen refrained from drawing any explicit conclusions on these findings.

Through initial baseline testing of the participant's hearing, Allen determined that he had average ability to perceive the distance of a sound. This was tested with resonating strings and a ticking watch. However, he was found to be extremely acute in his ability to hear very high‐ and low‐frequency sounds. The newly invented Galton's whistle which could be calibrated to test the limits of human hearing was used to determine this (Galton 1876). Allen conducted repeated testing of the participant's ability to discriminate different notes played on a piano or sung. The participant consistently failed to discern these differences and responded that they were “exactly alike.”

Allen's testing of pitch discrimination was subsequently refined to increase the interval between the notes in a graded fashion until the participant could report an awareness of a difference in pitch. In this manner, Allen determined the threshold for the participant's categorical perception of notes. By employing systematic and repeated testing of incrementally adjusted stimuli, Allen found that the participant could only discriminate between notes from different octaves. However, those in the middle octaves were better discriminated than those at the lower or higher octaves. Allen plotted his participant's performance on a graph for discrimination at three octaves below and above middle C. In a footnote, he was careful to state that this was an indicative graphic and that there were limitations to the interpretation of the quantitative analysis.

Allen (1878b) reported that in contrast to his difficulty with pitch discrimination, the participant had a sensitive appreciation of consonance and discord when tested using two moveable organ pipes. The participant appeared to be indifferent to their aesthetic qualities although he did acknowledge the beauty of individual tones created by striking a waterglass or church bells. The participant was also successful in recognizing popular songs, but Allen argued that this appeared to be based on the appreciation of timing rather than pitch.

In what appears to be a novel line of inquiry, Allen tested the participant's sensitivity to the emotional tone of a musical piece. He was innovative in using a forced‐choice labeling test. This assessment technique did not come into widespread use until the 1940s (Plantz et al. 2026). Allen required the participant to judge whether a passage of music was “lively, gay, bright, subdued, tender, solemn, or majestic.” The participant was successful in this task but did not appear to be sensitive to further gradations or changes within a particular piece. Allen contrasted this with the participant's acute appreciation of meter in poetry which it seems Allen viewed as a related function. Allen himself had a deep regard for poetry and wrote his own throughout his life (Morton 2005c). He included this additional variable as related to music appreciation, although he did not develop this point further.

In these various tests, Allen was gathering evidence of dissociations in the processing of auditory stimuli for music, speech, emotion, and aesthetic appreciation. While Allen's investigation was primarily focused on music perception, he also tested his participant's music production abilities although this was reported in less detail (Allen 1878b). The participant was found to be unable to sing a tune with accuracy of pitch or to repeat individual sung notes. Allen did not choose to explore his observation that the participant had an unexpressive speaking voice and other signs of limitations in spoken expression. This line of investigation would have further documented whether there was a difference between the production of pitch variation in song and speech and determined if the impaired perception of song melody was paralleled in the ability to perceive meter in poetry. Although such questions might occur to present‐day researchers, they were unlikely to arise in this context as is discussed below.

In Allen (1878b) discussion of his findings, he made the point that music is typically appreciated by the simultaneous analysis of pitch, intensity and timing. However, the pattern of his participant's responses to auditory stimuli suggested a dissociation between these. The individual was found to be severely impaired for pitch discrimination but had a well‐developed perception of intensity and timing. Allen considered that these strengths may have had a compensatory function. As for the cause of these difficulties, Allen considered whether his participant's note‐deafness might have its physiological source in the peripheral auditory system in the organs of Corti or in higher connections from each ear to the central nervous system. Allen's commentary reflected his detailed understanding of the auditory system as demonstrated in his earlier work (Allen 1877) discussed above. Allen stated that the physical source of note‐deafness could not be determined as there was no means to inspect the relevant anatomical tissue or trace the neurological connections to identify any individual variation in sufficient detail. Allen argued that its cause could only be determined when there was a means to investigate the individual physiological variation in the organs of Corti and the white matter fiber tracts ascending to the auditory cortex.

Finally, Allen extended an invitation to other investigators to come and observe the replication of his experimental results. He also urged other psychologists to find similar participants and collect additional data employing the same tasks and instruments that he did. In this way, Allen asserted the reliability, validity and rigor of his methods and encouraged the collection of further evidence to progress the understanding of individual variation in music processing. As will be discussed below, this invitation had had very limited uptake at the time. This lack of response from the research community is somewhat surprising since the contributors and readers of the journal *Mind* represented a large, significant and diverse group of leading figures in the science and philosophy of psychology (Staley 2009). Moreover, there was wide dissemination of Allen's study. The notice of Allen (1878b) publication appeared in leading British periodicals and the popular press including *Nature, The Athenaeum, The Independent, The Nation, Littel's Living Age, The Christian Union*. There were also notices in the leading French psychology journal *Revue Philosophique de la France et de étranger* and the German periodical *Zeitschrift für Philosophie und philosophische Kritik*.

After his study on note‐deafness, Allen returned to research on vision and aesthetics and employed observations or qualitative data to support his theoretical arguments. There does not appear to be further significant consideration of music in any of Allen's hundreds of publications (Morton 2005b). Although he continued to pursue his interest in other psychological processes, no other publication of his has been located with the same degree of systematic testing of perception. The study on note‐deafness stands out in his body of work for its use of experimental design and quantitative methods. Allen employed theoretical argumentation to frame his investigation and maintained an objective stance, offering minimal speculative comments on wider issues.

It is argued here that Allen's case study is a fundamental departure from the typical approach in contemporaneous psychological research which employed introspective analysis to describe conscious human experience (see Wilson 1999). It is more akin to the new style of psychophysical research which tested participants' responses to stimuli which had begun on the Continent in the late 1860s (Levelt 2013). Neary (2001) and Green (2009) plotted the development of empirical research in the field by examining the early issues of the journal *Mind*. However, Allen's article is not mentioned in either study. Green (2009) identified the later article on the ability of people to introspect on mental imagery by Galton (1880) as the first of this kind to be published in *Mind*. It is argued here that Allen's study should be given recognition as one of the first British investigations of perception using objective experimental methods.

## 5 Impact and Legacy

### 5.1 Immediate Responses to Allen (1878b) Work on Note‐Deafness

Allen's request for others to collect further evidence of note‐deafness had one immediate response. This research note and Allen's commentary on it appeared in the next issue of *Mind* (Simcox 1878). The author was the English philosopher Edith Simcox (1844–1901) who wrote on many topics that were reviewed in the journal. In contrast to Allen's study, Simcox provided an anecdotal introspective self‐report of her own note‐deafness. She reported being unable to distinguish two tones with intervals of less than a fifth. She described how she learned to play music as a child from memory but couldn't discern if the notes were correct. While her sense of tune was impaired, she reported having an average sense of time which she, like Allen, judged to be reflected in her average ability to compose metered verse. She discussed her poor ability to sing in tune and sing in harmony with others and questioned whether this was a “motor ataxia” rather than a perceptual problem. This suggestion is similar to Allen's consideration of his participant's difficulties in expression as arising from a motor source. These interpretations by Allen and Simcox point to different assumptions about the relation between perceptual and expressive processes that would be adopted in the late 1880s as discussed below.

Although her brief anecdotal report was of limited value, Simcox offered several suggestions for future empirical testing. First, she wondered whether a microphone would help amplify the stimuli and aid the perception of a difference between a third and a fifth. This instrument was a new invention at the time (Dolbear 1878). Her point addressed Allen's suggestion that intensity was judged independently from pitch and could be used to compensate for a difficulty in discriminating tones. Second, Simcox raised the question of whether some animal model might be pursued by vivisection to determine the physiological basis. In the mid‐1870s, London based physiologists investigated the cortical representation of auditory processing through experiments conducted on animals (Heffner 1987), however there were also growing public calls for banning the practice. Scientists lined up on both sides and Darwin was involved in the debate (Burkhardt and Secord 2015). However, it is unlikely Allen would have opted for such an approach given the biocentric tone of his other writings (Rodgers and Greenslade 2005).

Finally, Simcox remarked:

…but if the present high degree of musical sensibility is a development, and the primitive savage as insensible to musical intervals as Mr. Allen's subject and the present writer, it would probably be agreed that the change is more likely to have taken place in the elaboration of nervous sensibility than in the physical structure of the ear.

Simcox 1878, 403

This notion reflects contemporaneous views about variation in human evolution which Allen did not share, judging from his writings on color perception and color names discussed above. Rodgers and Greenslade (2005) describe Allen's view of human variation and its relationship to culture as comparative and relativist, akin to views expressed by the anthropologist Edward Burnett Tylor (1832–1917) in his book on *Primitive Culture* (1871). In his brief response to Simcox, Allen noted that her difficulties were not the same as the ones he observed in his participant and that there was a qualitative difference between them which was not to be attributed to degree of severity (Simcox 1878).

Rigorous testing of musical ability did not continue after Allen's singular attempt (see Humphreys 1993). While Galton had been involved in the testing of Allen's participant in 1878, he did not pursue empirical investigations of musical abilities or make use of Allen's tests in his later research (e.g., Galton 1883). It does not appear that Allen collaborated with Galton after 1878. Although Galton continued to be interested in the unequal distribution of musical talent in people, his investigations were based on self‐report and the measurement of basic aspects of hearing (e.g., the range of pitches that were audible with his whistle). He also held the belief that sensory discrimination abilities were indicative of an individual's intelligence. In contrast, Allen had made it clear that his note‐deaf participant was highly intelligent, and his perceptual difficulty could not be accounted for in this way.

Allen (1878b) original work on note‐deafness did figure in a significant early initiative in British psychology. Joseph Jacobs (1854–1916), an anthropologist who studied under Galton, wrote an article in the journal *Mind* calling for the formation of an experimental psychology society (Jacobs 1886). He listed several aims and objectives of such a research group. One of the topics that he specifically singled out for investigation was note‐deafness. This can be taken as evidence that there was awareness in the wider intellectual community of the significance and value of Allen's experimental research into individual variation in music perception that persisted for several years after its publication.

From further afield, the American art critic Mariana Alley Griswold Van Rensselaer (1851–1934) referred to Allen's study (1878b) in a call for universal training in music for children. She made an exception for “those hopeless ones afflicted with the just realized deficiency called ‘note deafness’” (Rensselaer 1880, 385). This echoes Allen's point that exposure and educational opportunity have little effect on this physiological impairment. Beyond these few examples, the current investigation has found that Allen's research did not lead to further consideration of variation in human auditory perception immediately following its publication. The reasons for this are examined below.

### 5.2 Developing Psychophysiological Models of Music

There is a sense in which Allen's work can be viewed as a premature scientific discovery due to a lack of canonical knowledge to connect it to (see Hook 2002). In addition to limited understanding of the neurophysiology of audition at the time, there was also little development of a psychophysiological model of the expression and perception of music to motivate the line of research Allen had attempted to initiate. The description of acquired amusia through brain injury in adults was not made until later in the century (Graziano and Johnson 2015). Therefore, Allen himself and others who read his article in 1878 could not connect his observations of congenital music processing difficulties to selective acquired neurological impairments in music. While the German physician Adolf Kussmaul (1822–1902) did include a pathway for music perception in his model of language processing based on clinical and pathological observations in 1877, there was little development in the study of acquired impairments of music until at least a decade later (Johnson and Graziano 2003). This lacuna reflected the lack of integration between clinical and academic research in the field of psychology in the 1870s. This issue is referred to directly by James Crichton‐Brown (1840–1938) (1878) in his Presidential Address to the Medico‐Psychological Association. He enumerated recent advances in the field in comparative psychology, mental evolution, language, and “the characteristics of those born devoid of particular senses” (Crichton‐Browne 1878, 354). He identified significant work by Spencer, Bain, Darwin, Galton, Romanes and “…by Mr Grant Allen who is delineating the foundations of our aesthetic perceptions and feelings” (Crichton‐Browne 1878, 354)

One of the few citations of Allen's study in the clinical literature appeared several decades later in the English physician Frederick Bateman's (1824–1904) review of evidence on the localization of language (Bateman 1890). He considered the evidence from neurological cases in which the perception of musical sounds was impaired alongside the perception of speech. In discussing the nature of acquired sensory aphasia which involves a difficulty in the understanding of spoken language, Bateman reproduced the full details of Allen's case. However, he made no comment about the fact that Allen's case study was of a healthy individual with a developmental difficulty rather than a known neurological disease. Moreover, a report in the *British Medical Journal* (1894) used Allen's term note‐deafness in discussing the dissociations observed in patients with various combinations of acquired aphasic and amusic symptoms.

The wider consideration of developmental language disorders alongside acquired language disorders also began in the 1890s (Lorch and Hellal 2012). The term “amusia” began to be used at this time to specifically refer to an acquired neurological disorder in music processing that corresponded to aphasia (e.g., Knoblauch 1890). The developmental equivalent of “congenital amusia” appears around the same time. The introduction of this new terminology is indicative of the significant efforts in the study of music and language processes at the end of the century. One notable synthesis of the evidence regarding the psychological representation and function of music and language can be seen in the article *Internal Speech and Song* (1893) by the American psychologist James Mark Baldwin (1861–1934). Baldwin drew on observations from healthy development, developmental difficulties and acquired neuropathological cases that involved language, speech and music. He presented a detailed consideration of the neurological and psychological processes underpinning the perception of music:

It is probable, accordingly, that there is a brain‐center for tune memories—a center whose impairment produces so‐called *notal amusia* ‐that this center is a part, in function, at least, if not anatomically, of the auditory center, and that cases will occur in different persons, of partial amusia, due to the degree in which this function involves others.

Baldwin 1893, 398

In developing this point, Balwin directly cited Allen's case (1878b). This indicates that there was continuing awareness of the significance of this case as evidence to support conceptualizations of music processing later in the 19th century. The major psychology textbook written at the time also features the details of Allen's contribution. The American philosopher George Trumbull Ladd (1842–1921) described tone deafness as a condition that occurs in people who are otherwise normal and likened it to color blindness as had been initially proposed by Allen (Ladd 1894). Ladd also pointed out that in current society this impairment did not interfere with a successful career except in music. However, he argued that if everything in society were dependent on this skill, many would be considered severely disabled. Almost a century later, the American neurologist Norman Geschwind (1926–1984) made a similar argument to that put forward by Ladd in his consideration of “dysmusia” (Geschwind 1984). He pointed out a large number of unknowns about this developmental deficit which underscores the lack of neuropsychological research into music to advance understanding of how it is processed up to the 1980s.

### 5.3 The Current Status of Allen's Case of Note‐Deafness

Over 80 of the 90 citations of Allen (1878b) case study as captured by Google Scholar are from 21st century publications. These are primarily works in current cognitive science research rather than 19th century history. This reflects a new high degree of interest in music processing, which had long been a marginal topic of research. In most of these recent publications, Allen's study is generally simply cited (sometimes with his name erroneously hyphenated) as the earliest description of congenital amusia without any further commentary. His investigation is often referred to as an anecdotal report which is then contrasted with present‐day systematic investigations. This suggests a lack of engagement with Allen's 1878 text. One notable exception to the superficial treatment of Allen's contribution by 21st century scholars was offered by the neurologist Oliver Sacks (1933–2015). He argued that this “superb” case study “established experimental neurology and psychology in the latter part of the nineteenth century” (Sacks 2008, 113). The detailed evidence presented here demonstrates that Allen's work was systematic and identified many novel avenues of research which were subsequently pursued much later on as discussed below.

Allen's original contribution about note‐deafness was not considered one of his lasting achievements. Although he was renowned for his fiction and nonfiction writings at the time of his death, the numerous obituaries and memorials do not make mention of his publication on note‐deafness. An extensive bibliography which appeared at the time of Allen's death does not include it (Clodd 1900). Allen's work in psychology has attracted a small degree of scholarly interest more recently, but this does not include consideration of his contribution (Allen 1878b) to the study of musical ability. For example, there is no commentary about this work in Morton's lengthy biography even though there is detailed treatment of Allen's scientific activities in the period from 1876 to 1879 (Morton 2005c). In recent commentaries, Allen is typically portrayed as a generalist and popularizer of science (e.g., Mills 2021). As demonstrated here, his study of note‐deafness is direct evidence of his abilities as an insightful and methodical empirical researcher.

### 5.4 Lack of Impact

After his single case study, it appears he did not carry out further experimental psychology studies or returned to the topic of musical ability in his more discursive writings. The research which Allen initiated on note‐deafness was not pursued further by him and there was limited propagation of his contribution in subsequent decades. There are several personal circumstances which may account for the lack of uptake of this line of research. One issue was Allen's lack of financial support, which constrained his ability to pursue psychological research despite the recognition he gained for his early efforts. His evolutionary work on perception in the 1870s had won him respect from many leading intellectuals of the day. Darwin had copies of both of Allen's books (1877, 1879) as well as the German translation of the *Colour‐Sense (Der Farbensinn*) (Allen 1880) and Darwin's marginalia suggests that he read them carefully (Gregorio and di 1990). Spencer, Wallace and the biologist Thomas Henry Huxley (1825–1895) were also high‐profile supporters, indicating that Allen had established himself with intelligentsia in the social network of Cambridge, Oxford and London. However, he did not secure one of the rare university posts to ensure his livelihood (Morton 2005c). Unlike his peer Romanes and more senior mentors, he did not have the advantage of inherited wealth and needed a source of income to support his wife and son (born July 1878). This lack of financial support and institutional resources limited his ability to continue to carry out experimental research. In the 1880s, Allen gave up his hope of gaining an income through science to concentrate his primary energies on the more lucrative business of fiction (Allen 1894). At this point, Allen's career trajectory took him on a different path, and he became a prolific writer of novels and short stories. Nevertheless, he continued to regularly publish articles on a broad range of scientific topics and maintained his intellectual social network (Morton 2005a, 2005b).

Allen did continue to write extensively on evolution, aesthetics and psychology. These articles were intended for a “popular” audience but contained solid empirically based observations and high‐quality source material. One interesting example of this is Allen's article “A Thinking Machine” which appeared in *The Gentleman's Magazine* (Allen 1886). He put forward a well‐evidenced argument about the dynamic brain connections for representing words and thoughts. In this article, he pointed out that localizing the pathological source of a selective impairment is not the same as localizing its function. This echoes a point made by London neurologist John Hughlings Jackson (1874) (1835–1911). Allen also cited the French psychologist Théodule Ribot's (1839–1916) work on disorders of memory (Ribot 1882). These examples indicate that Allen maintained an advanced level of informed scholarship and continued to develop his thinking on matters of human psychology.

Allen's continuing research in to other areas of science were also recognized as significant. Darwin, Spencer and Huxley all praised his writing on biology. An obituary of Allen by the English naturalist Herbert W. Tompkins (1867‐?) (1904) claimed that Allen's 100 writings on botany represented the largest body of knowledge on the subject. Furthermore, Tompkins asserted that Allen's presentation of the complexity of issues in science was on a par with Huxley's. In fact, Allen co‐authored a 40‐page review on “A half century of science” with him (Huxley and Allen 1886). However, Allen's view of evolution was distinct from these other thinkers. His ideas about descent with modification included both genetic and cultural sources. He was not a Lamarckian, rather he argued that things were passed down from generation to generation both through biological mechanisms and cultural institutions. This latter argument can be seen in Allen's summary of the progress of science from 1836 to 1886 in the Jubilee number of the *Fortnightly Review* (Allen 1887).

In psychology, the evolutionary impulse has mainly manifested itself in Herbert Spencer, and to a less degree in Bain, Sully, Romanes, Croom Robertson, and others of their school. The development of mind in man and animal has been traced *pari passu* with the development of the material organism. Instinct has been clearly separated from reason: the working of intelligence and of moral feeling has been recognized in horse and dog, in elephant and parrot, in bee and ant, in snail and spider. The genesis and differentiation of nervous systems have been fully worked out. Here Maudsley has carried the practical implications of the new psychology into the domain of mental pathology, and Ferrier has thrown a first ray of light upon the specific functions of portions of the brain. Galton's “Hereditary Genius” and other works have also profoundly influenced the thought of the epoch: while Bastian, Clifford, Jevons, and others have carried the same impulse with marked success into allied lines of psychological research.

Allen 1887, 857

These selected publications of Allen's evidence the substantial position he held in the 1870s and 1880s as a significant contributor and commentator on human behavior.

### 5.5 Ongoing Research Issues Initially Identified by Allen

There has been continuing investigation of many questions Allen raised in the 19th century. Allen (1878b) had suggested that note‐deafness was a rare occurrence similar to color blindness. He also argued that the difficulties his participant had were qualitatively different from others who were simply unmusical. The frequency in the population, patterns of inheritance and the degree of variation in music perception are still not yet fully determined. Half century after Allen's (1878b) study, consideration of the relation between tone deafness and color blindness was raised again. The Australian Railway Medical Officer George Henry Taylor (dates unknown) stated that Mendel's Law is in operation for color blindness and tone deafness at similar frequencies of occurrence (Taylor 1924). However, he observed that the two do not co‐occur in one individual and while color blindness seems to be inherited through men, tone deafness may occur in women. A recent twin study found that musical pitch recognition was over 70% heritable (Drayna et al. 2001). However, there is still a question about whether congenital amusia is at the tail end of a spectrum of ability or is a distinct condition. Co‐occurring difficulties in various aspects of sound processing and related speech perception and production are also currently being actively pursued (Zatorre et al. 2002). The interplay between inherited characteristics and cultural influences and how they might shape individual variation was raised by Allen in his perception research. How these different factors shape specific disabilities is a live topic of inquiry in neuroscience and philosophy today (Stoner 2016).

Allen (1878b) had pointed out that the neurological basis of note‐deafness could not be determined due to a lack of investigative tools. He suggested that this would have to wait for future developments. In fact, there was no method for determining this until quite recently (Glickstein 2007). Nevertheless, the peripheral and central nervous system mechanisms for human pitch perception are still poorly understood (Yost 2009). Recent efforts to identify the neurological basis of difficulties with music processing have not been definitive (Chen and Yuan 2016). There is an ongoing debate about the neurophysiology underlying the pitch perception impairment in congenital amusia. One recent study found that regions of the auditory cortex that respond to pitch in amusic individuals were not different from controls (Norman‐Haignere et al. 2016). Other evidence that indicates the source may be in variation in the white matter connectivity between the auditory and frontal cortex (Loui et al. 2009; Wang et al. 2017). These findings suggest that the physiological basis of congenital amusia may be due to a central rather than peripheral impairment as Allen suspected but it appears that its source has still not been fully determined. There are also recent suggestions that tone‐deafness is one of several different phenotypes reflecting cortical processing variations and that congenital amusia may be co‐morbid with developmental dyslexia and/or prosopagnosia in some individuals (Couvignou et al. 2023; Paquette et al. 2018). This research into people with music processing difficulties and co‐occurrence of other developmental patterns of impairment continues to broaden our understanding of different cognitive abilities which have a culturally determined value.

Allen's (1878b) testing of his participant's emotional response to music was also a significant innovation. This aspect has only begun to be considered under the term musical anhedonia in the 21st century (Satoh et al. 2011). More broadly, there is new appreciation for Allen's groundbreaking work on physiological aesthetics (1877), which served as a framework for the case study under consideration here. He has recently been identified as the founder of the field of evolutionary aesthetics, which is now a growing topic of interest (Kiianlinna 2023).

## 6 Concluding Remarks

This article argues that the procedures Allen employed in his investigation of his participant's pitch perception would be recognized as best practice today and his research questions are still not fully answered. It is a significant contribution that has been neglected. The present examination of Allen's 1878 case study of congenital amusia clearly demonstrates the systematic, rigorous and objective data gathering that he used to document various dimensions of his participant's musical processing difficulties. He detailed his participant's normal hearing, general intelligence, advanced level of education and lack of ability to appreciate or produce music despite motivation and training. He also documented other family members' music processing difficulties. This addressed the possible sources for the music processing impairment from both nature and nurture.

In the design of his tests, Allen (1878b) addressed several focused research questions about musical ability and its relationship to other aspects of auditory processing and oral expression. He created experimental tasks to determine the involvement of various components of music perception and recognition and the relationship between the perceptual problems in music and those in potentially related domains. He objectively investigated the participant's responses to individual tones, musical passages, connected speech, and environmental sounds. These investigations were conducted to ascertain the degree of auditory ability in perception and expression and related aesthetic and emotional components. His report demonstrates a systematic approach to testing and the analysis of results, while his interpretation of findings was cautious and careful and avoided speculation. It is argued here that his study represents one of the earliest English detailed experiments in psychology. Allen raised novel research questions about the relation between music perception and production and between music and speech processing. Many of these questions could not be answered in the 1870s and remain undetermined today.

Allen (1878b) had drawn an analogy between note‐deafness and color blindness. At the time he was working, the latter was understood to be a rare inherited difficulty in color perception in otherwise typically developing people. There were significant socio‐economic drivers for the development of assessment techniques and the identification of epidemiological factors for color blindness (Bailkin 2005). While musical talent was a culturally valued personal ability in Victorian England (L. Jackson 2019), lack of it did not have similar consequences. As Allen's case was purported to be the first description, it was unknown how rare this condition was, whether it was inherited, and if it was due to a sensory impairment like color blindness. In this sense, Allen was a pioneer investigating novel and significant questions about the basis of human behavioral variation. Although his case study was published in a highly regarded journal and he had a strong personal network of powerful academic supporters including Darwin and Spencer, Allen's groundbreaking research was pursued by few others in subsequent decades. While Allen (1878b) study has now begun to be cited in the 21st century, there has been little detailed consideration of the case or his underlying theoretical framework, research questions, and interpretation of findings until the present investigation.

Allen (1878b) also raised novel questions about the subjective aesthetic and emotional experiences of people who have music perceptual impairments. This was part of his larger consideration of sensory responses to the environment and its role in evolution (Allen 1877, 1879). Evolutionists such as Darwin and Spencer held the view that music is a universal human capacity which serves as the basis for language. Allen's significant contribution was his questioning of how music and other perceptual abilities are manifested and subject to wide cultural influences and variation which form human aesthetic experiences.

## Disclosure

The author has nothing to report.

## Conflicts of Interest

The author declares no conflicts of interest.

## Data Availability

Data sharing is not applicable to this article as no datasets were generated or analyzed during the current study.
